# Determination of the effect of abiotic stress on the oxidative potential of edible potato tubers

**DOI:** 10.1038/s41598-023-35576-9

**Published:** 2023-06-20

**Authors:** Elżbieta Wszelaczyńska, Jarosław Pobereżny, Katarzyna Gościnna, Małgorzata Szczepanek, Magdalena Tomaszewska-Sowa, Grzegorz Lemańczyk, Karol Lisiecki, Cezary Trawczyński, Dominika Boguszewska-Mańkowska, Milena Pietraszko

**Affiliations:** 1 Institute of Agri-Foodstuff Commodity, Bydgoszcz University of Science and Technology, 7 Kaliskiego St., 85-796 Bydgoszcz, Poland; 2 Department of Agronomy, Bydgoszcz University of Science and Technology, 7 Kaliskiego St., 85-796 Bydgoszcz, Poland; 3 Department of Agricultural Biotechnology, Bydgoszcz University of Science and Technology, 6 Bernardyńska St., 85-029 Bydgoszcz, Poland; 4 Department of Biology and Plant Protection, Bydgoszcz University of Science and Technology, 7 Kaliskiego St., 85-796 Bydgoszcz, Poland; 5 Potato Agronomy Department, Plant Breeding and Acclimatization Institute, National Research Institute, 05-140 Jadwisin, Poland

**Keywords:** Environmental chemistry, Chemical biology, Physiology, Plant sciences

## Abstract

Stress factors occurring during the growing season and potato storage, can negatively affect the quality of tubers, including an increased tendency to enzymatic darkening. Abiotic stress due to water shortage is a major factor limiting agricultural production. The purpose of the study was to determine the effect of cultivation technology based on the use of biostimulant, hydrogel and irrigation as well as storage on the propensity to darkening and the content of sugars and organic acids. The results show that genotypic and technological variability in interaction with growing season conditions had a significant (*p* < 0.05) effect on the oxidative potential (OP) of potato tubers. The Denar cultivar, compared to the ‘Gardena’, was characterized by a lower tendency to enzymatic darkening. Application of biostimulant and hydrogel generally contributed to lowering the oxidative potential of the tested cultivars. The application of anti-stress agents had no effect on organic acid content. The long-term storage caused an increase in the content of total sugars (TS) (22%), reducing sugars (RS) (49%), chlorogenic acid (ACH) (11%), and loss of ascorbic acid (AA) (6%) in the tubers which contributed to an increase in the oxidative potential of potato tubers (16%). The correlation coefficients obtained (*p* < 0.05) confirm the dependence of OP on the concentration of organic acids.

## Introduction

The potato (*Solanum tuberosum L.*) is an integral part of the global food system^1–4^. Its position among arable crops is due to its high production of usable biomass per unit area, its wide range of uses, and the high nutritional value of the tubers^1,5–7^.

Table potatoes are perceived through visual sensations. The appearance of the tubers is a function of commercial grading and the purchasing decision^8–10^. Any adverse changes in the external and internal appearance may determine the volume of potato supply on the market^11,12^. The most common changes include the discoloration of the tuber flesh. As discoloration can occur at any stage of production and processing, this is one of the main challenges faced by the potato industry^9,13^.

The process of raw tuber darkening results from the oxidation of phenolic substrates such as tyrosine, chlorogenic acid (ACH), and caffeic acid to quinones in the presence of the enzyme polyphenol oxidase (PPO). Quinones form final products in the form of stable brown, black, and red melanin^9,14^. The discoloration of raw potatoes is a crucial issue, as enzymatic darkening is the second most significant factor in the degradation of potato quality^4,15^. The intensity and rate of the enzymatic darkening process are mainly determined by the activity of the enzyme (PPO) and the content in the tubers of bioactive components such as ascorbic acid (AA), citric acid (AC), polyphenols, and, primarily, ACH, which accounts for approximately 90% of all the phenolic compounds found in potatoes^16^. It should be noted, however, that AA and AC are among the compounds that prevent enzymatic discoloration^9,17^. Other tuber components, such as sugars or amino acids, are also involved in the discoloration reaction, which reduces the nutritional value of the tubers^5,18,19^. The content of the above-mentioned compounds is, to a large extent, a genetic trait. However, the edaphic factors such as environmental conditions, cultivation technology type, and stresses occurring during plant growth also have a major influence^9,20–22^.

Due to their shallow root system, potatoes are plants that are very sensitive to water shortages during the growing season^7,22–24^. The occurrence of water stress reduces the tuber quality and increases the intensity of the enzymatic darkening process in the flesh, as it increases the tyrosine content in the tubers^17,25^.

Of the total potato production, approximately 95% is destined for long-term storage of between 1 and 9 months. This period is the most difficult stage in the potato production cycle. Many authors^26,27^ are of the opinion that a failure to maintain a constant temperature and relative air humidity, recommended for the market destination, results in the deterioration of tuber quality in terms of chemical content and susceptibility to darkening.

There is a need for continuous plant breeding work and the introduction of modifications to the existing cultivation and storage technologies in order to produce varieties that are more resistant to stress factors^28^. For this reason, new potato cultivars are being successfully introduced to world markets, and cultivation technologies are being adapted to sustainable farming practices. It is important for the increase in the quality of the final product to be combined with maximising output per area unit while minimising the environmental impact^29–31^. During the potato cultivation period, new-generation stress-preventing preparations are introduced, and water is replenished during times of shortage^10,22,23,25^.

The hypothesis predicts that changes in cultivation technologies involving the use of anti-stress preparations and water replenishment during periods of shortage would contribute to reducing the value of the oxidation potential (OP) of the table cultivar potato tubers. In addition, the blackening process of the tuber flesh of table potatoes cultivated under a system using anti-stress substances and irrigation has not been sufficiently investigated to date. The problem has also not been sufficiently examined as regards long-term potato storage.

Therefore, this study was conducted in order to identify the effects of cultivation technology based on the application of a biostimulant, hydrogel and irrigation, as well as storage, on the susceptibility to darkening and the sugar and organic acid contents.

## Results and discussion

**Figure 1:**
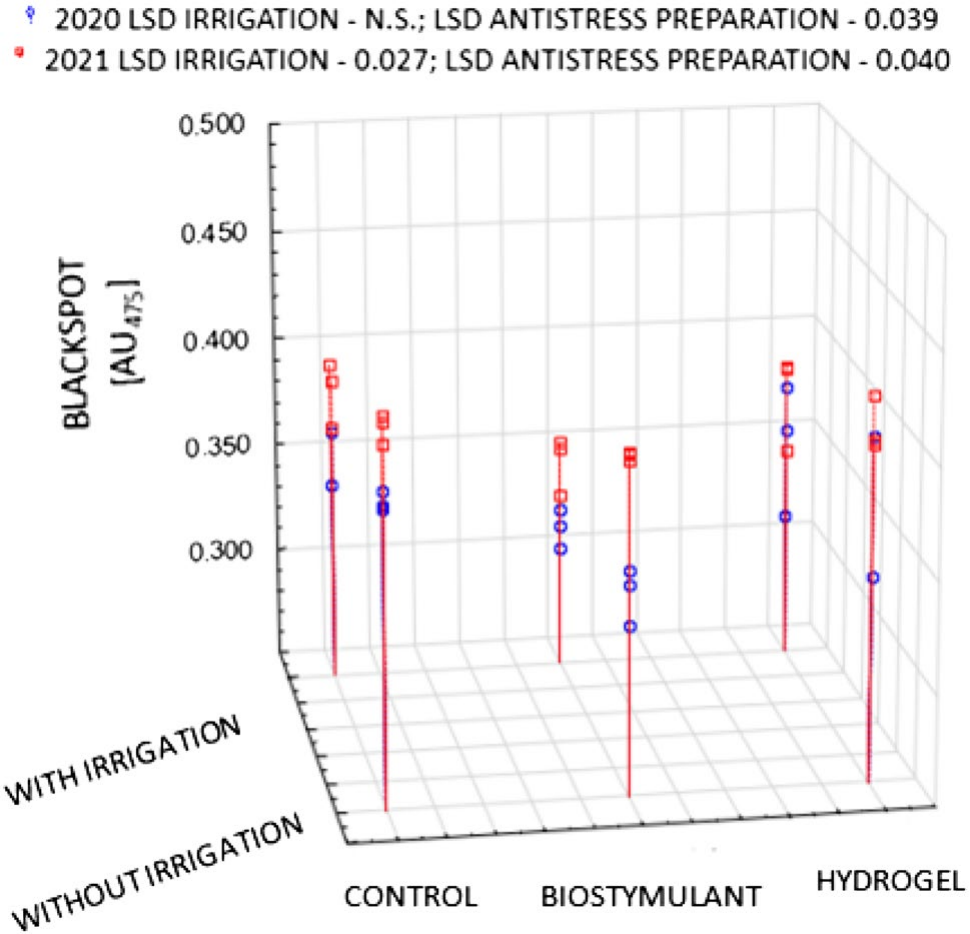
Post-harvest OP (blackspot) of Gardena cultivar in 2020 and 2021 crop years depending on irrigation and type of stress-reducing preparations.

**Figure 2:**
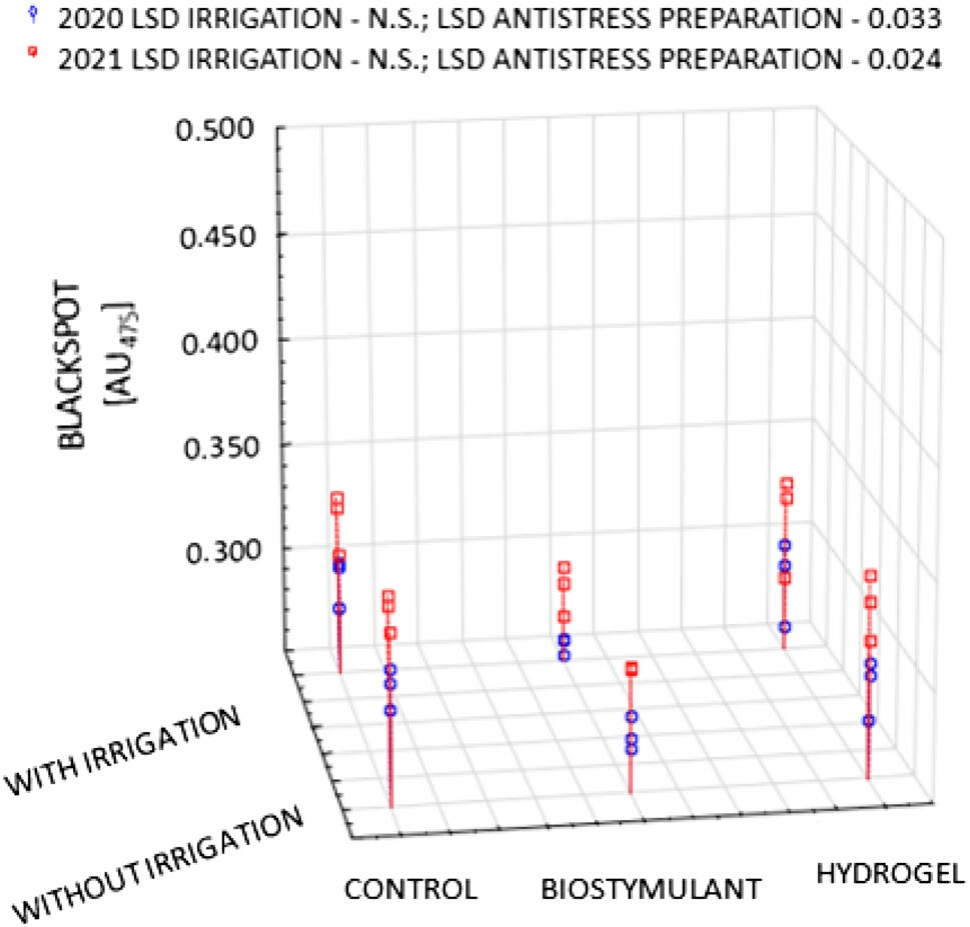
Post-harvest OP (blackspot) of Denar cultivar in 2020 and 2021 crop years depending on irrigation and type of stress-reducing preparations.

**Figure 3:**
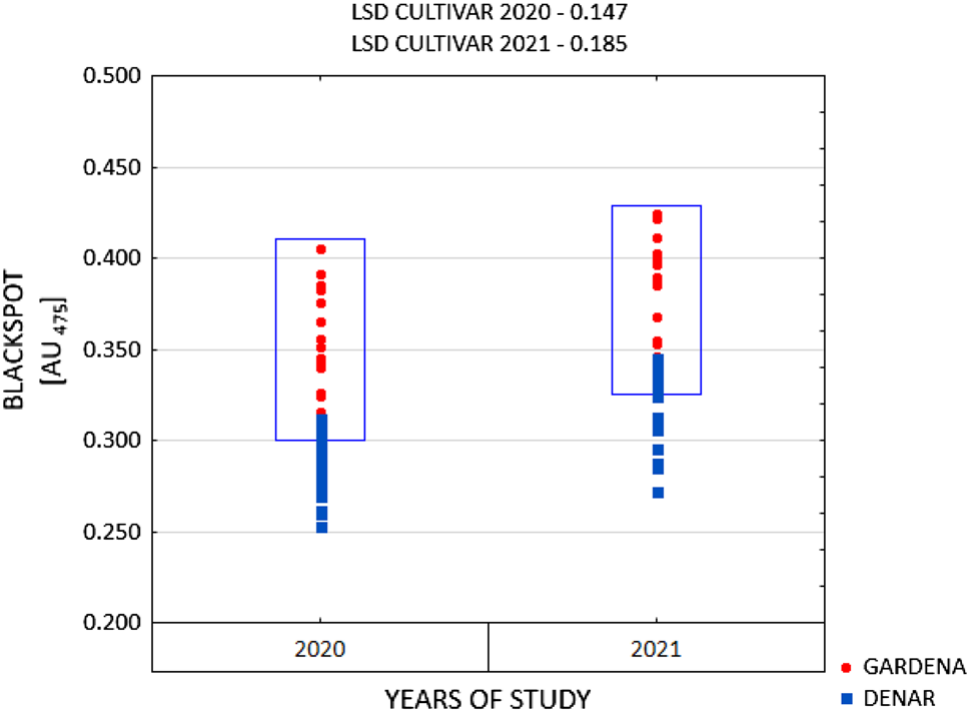
Post-harvest OP (blackspot) of potato tubers of Gardena and Denar cultivars depending on the crop years.

One of the major characteristics determining the sensory quality of potato tubers intended for direct consumption is the susceptibility to raw flesh darkening. Immediately after the harvest, irrespective of the years and experimental factors, the medium-early Gardena cultivar was characterised by a higher value of the OP (blackspot) of 0.371 AU_475_, as compared to that of the early Denar cultivar of 0.301 AU_475_ (Figs. 1 and 2). However, according to the classification by (Table 1)^32^, the tubers of both the Gardena and Denar cultivars can be regarded as moderately resistant to enzymatic darkening. The functional value of potatoes, including susceptibility to enzymatic darkening, is determined to a large extent by genetic determinants and, therefore, by the earliness group^9,16,33,34^. Pobereżny et al.^16^ noted that tubers from cultivars with the same market destination, but belonging to the same earliness group, can have varying levels of susceptibility to darkening in their flesh. Other authors^15,35–38^ suggest that the intensification of the potato tuber enzymatic browning process is also linked to the occurrence of abiotic stresses. This shows that the susceptibility to darkening of the tuber flesh does not only depend on genetic determinants but also on cultivation conditions^39–41^. The flesh of the cultivars whose tubers are exposed to stress for a longer period may show a greater susceptibility to darkening. The Gardena and Denar cultivars under study differed in the magnitude of the OP in particular years, which indicates the varied response of the cultivars to weather conditions in the years of cultivation (Figs. 1 and 2). The correlation between the cultivars and the weather conditions was also demonstrated by other researchers^38,42^. It was pointed out that in the current study, a higher value of the OP was exhibited by the tubers from 2021, which was less favourable to potato cultivation (Fig. 3). Bienia et al.^42^ demonstrated in a three-year study that darkening of the raw tuber flesh was determined by the cultivar and weather conditions during the years of the study. The flesh of the tubers cultivated in the year with exceptionally dry July and August, and moist June and September, darkened the most. In the two-year period of the current study, the year 2021 was characterised by a lower amount of precipitation as compared to 2020. It should be noted, however, that the precipitation distribution was less favourable than that in 2020. Many authors^9,38,43^ emphasise that the preservation of the light colour of the flesh of raw tubers is promoted by sunny weather with a favourable precipitation pattern throughout the growing season. Bienia et al.^42^ emphasise that despite the proven statistical differences in the susceptibility to enzymatic darkening, the variability of this trait was very low, which is consistent with the results of the current study.

**Table 1:** Classes of susceptibility to oxidation darkening processes, according to Dean et al^32^.

Susceptibility classes	Colorimetric method [AU_475_]
1. Resistant to darkening processes	0–0.200
2. Moderately resistant to darkening processes	0.210–0.400
3. Moderately susceptible to darkening processes	0.410–0.600
4. Susceptible to darkening processes	0.610–0.800
5. Very susceptible to darkening processes	> 0.800

Sawicka et al.^43^, and Hussain et al.^9^ report that the dominant factor that influences the susceptibility to enzymatic darkening is the conditions of the growing season in interaction with not only genotypic but also technological variability. In the current study, the application of irrigation during the plant growing season reduced the potential oxidative value immediately after the harvest, although this was generally not statistically proven. A reduction in the potential value for the Gardena cultivar was, on average, by 8.5% (and for the Denar cultivar by 4.9%) as compared to non-irrigated tubers (Figs. 1 and 2). The positive effect of the biostimulant was also proven. The application of the biostimulant on the irrigated plots significantly reduced the susceptibility to enzymatic darkening as compared to the control (Figs. 1 and 2), while for the non-irrigated tubers, this relationship was not so unambiguous. This is in line with the results of previous research into enzymatic darkening of not only table cultivars but also those intended for processing (fries and crisps)^24,44^. Biostimulant (BioAlgeen S90) in its composition contains higher amounts of micronutrients, which increase the efficiency of the plants' uptake of macronutrients, including nitrogen. This can lead to an increase in enzymatic darkening of potato tubers as indicated by the results of a study by Bienia et al.^42^.

In a study by Zarzecka et al.^38^, the application of the UGmax resulted in little variation in the darkening of the raw tubers of table cultivars, as it only indicated a tendency to the detriment of the control variant. On the other hand, Kołodziejczyk^45^ and Zarzecka et al.^38^ noted no significant effect of the UGmax on the intensity of raw tuber flesh darkening. At the same time, the current study achieved a similar effect following the application of hydrogel on both irrigated and non-irrigated plots (Figs. 1 and 2).

Susceptibility to enzymatic darkening depends on the chemical composition of the tubers, which is determined genetically but can also be modified by edaphic factors or storage conditions and duration^14,16,34,44^.

**Figure 4:**
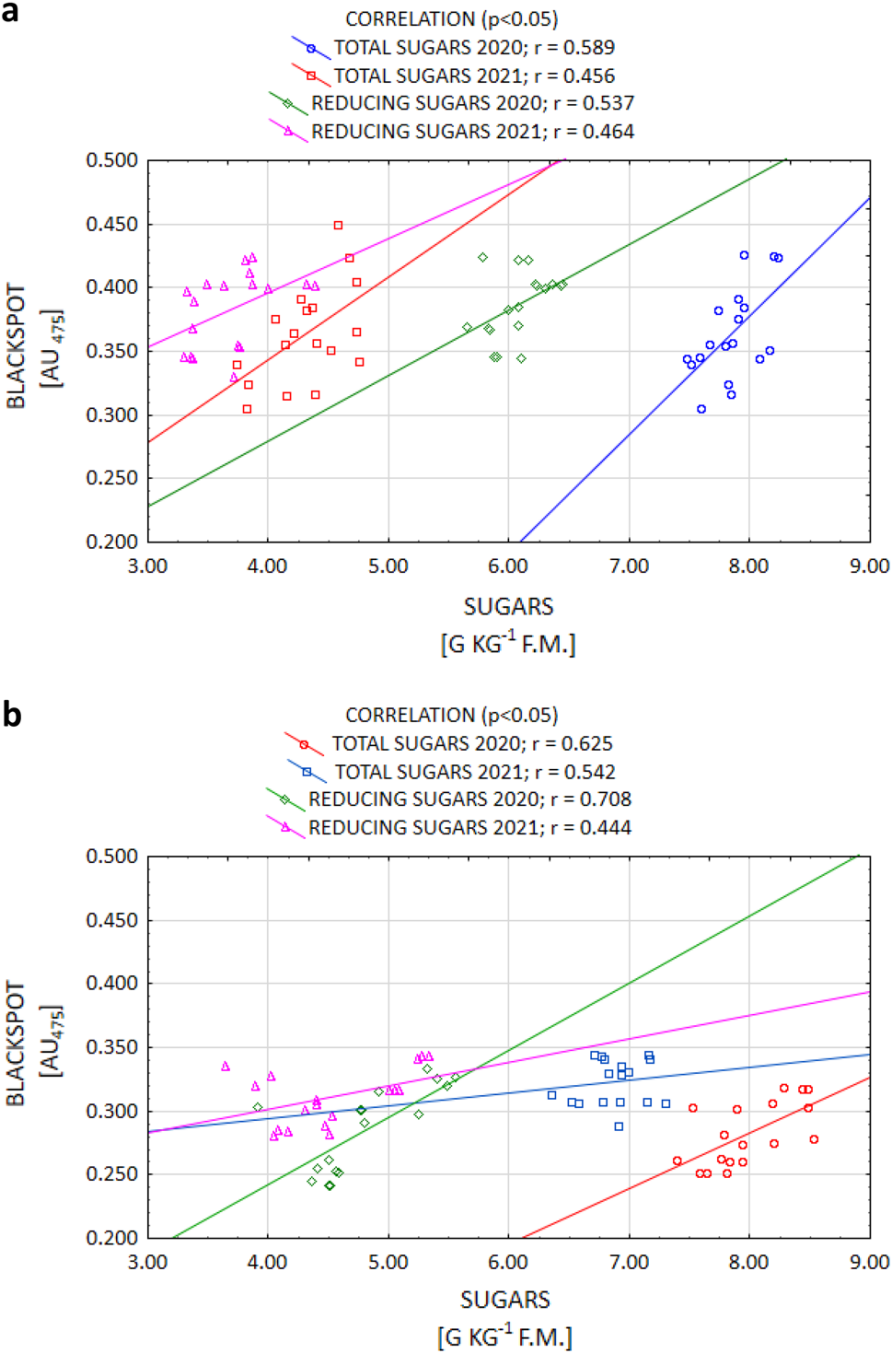
Post-harvest correlation between OP (blackspot) and the content of TS and RS of Gardena (**a**) and Denar (**b**) cultivars in 2020 and 2021 crop years.

One factor that largely influences the enzymatic darkening of the potato tuber flesh is the sugar content. The flesh of potato tubers containing a higher proportion of RS is subject to more intense non-enzymatic browning processes during heating^17,25,46^. At elevated temperatures, the RS contained in potatoes form amino sugars which, as a result of biochemical transformations, leads to the formation of melanoidins that give the tubers their brown colour^3,47^. In the current study, the OP value was determined by the TS and RS content for both the Gardena and Denar cultivars, which is indicated by the correlation coefficients at a level *p* < 0.05 (Fig. 4a,b).

Regardless of the factors used, in each year of the study, the early cultivar Denar was characterised by a higher content of TS and RS as compared to the medium-early cultivar Gardena (Table 2). The ability to accumulate sugars in potato tubers is a varietal trait, and the cultivars with a shorter growing season accumulate more sugars than the cultivars with a longer growing season^3,33,46,48^. The application of the biostimulant, hydrogel, and irrigation during the growing season significantly modified the TS and RS content in the tubers immediately after the harvest (Table 2). The application of stress-preventing preparations in the form of a biostimulant and hydrogel increased the TS and RS content in the tubers as compared to the control. However, this was generally not statistically proven on both the irrigated and non-irrigated plots (Table 2). Previous studies conducted by multiple authors^49–51^ demonstrated that the application of biostimulants (Asahi SL, Bio-Algeen S-90, Kelpak SL and Trifender WP) and hydrogel in the cultivation of root crops increases the content of both TS and RS. However, the magnitude of the effects of the application of these preparations is determined by the preparation type and dose, and for the biostimulant, also by the frequency of application^51,52^. Wszelaczyńska et al.^50^ achieved a slight increase in the TS and RS content by using biostimulant (Kelpak SL) in carrot cultivation. On the other hand, Kulikowski et al.^49^, by applying hydrogel in the cultivation of sugar beet, achieved an increase in the TS content in the roots by up to 44%. The current study noted that in 2021, which was less favourable to potato cultivation, the effects of the biostimulant and hydrogel were stronger as compared to those in the year 2020 (Table 2). This is related to the distribution of temperatures and precipitation during the potato growth period, as the year 2021 was characterised by a greater water deficit at higher temperatures during the periods critical for the potato, i.e. flowering and tuber setting. By absorbing very large amounts of water, hydrogels increase water-holding capacity and soil retention, thus preventing water stresses by providing plants with moisture. Moreover, these preparations effectively reduce the evaporation of water from the soil, and their performance is greatest on light soils used for potato cultivation^31,49,53^. In addition, irrespective of the year of the study, a clearly more beneficial effect of the biostimulant was demonstrated, as compared to the effects of the hydrogel. Environmental stresses are known to have a major impact on carbohydrate metabolism in plants^25,54^. However, the application of biostimulants (Asahi SL, Bio-Algeen S-90, Kelpak SL and Trifender WP) results in the normalisation of biochemical processes in plants by RS the effects of stress^31,51,52^.

**Table 2:** Post-harvest content of TS and RS of Gardena and Denar cultivars depending on irrigation and type of stress-reducing preparation.

Cultivar	Irrigation	Anti-stress preparation	Total sugars ([g kg^−1^ f.m.)		Reducing sugars (g kg^−1^ f.m.)	
			2020	2021	2020	2021
Gardena	Without irrigation	Control	7.86 ± 0.11*BC	6.01 ± 0.20B	4.01 ± 0.35A	3.27 ± 0.09A
		Biostymulant	8.03 ± 0.16CD	6.40 ± 0.08C	4.73 ± 0.41C	3.57 ± 0.37ABC
		Hydrogel	8.13 ± 0.15D	6.27 ± 0.08C	4.57 ± 0.24BC	3.39 ± 0.18AB
	**Mean without irrigation**		**8.01 ± 0.12**	**6.23 ± 0.20**	**4.44 ± 0.38**	**3.41 ± 0.15**
	With irrigation	Control	7.52 ± 0.05A	5.77 ± 0.10A	3.94 ± 0.32A	3.66 ± 0.19BC
		Biostymulant	7.75 ± 0.13B	6.05 ± 0.04B	4.19 ± 0.31AB	4.16 ± 0.20D
		Hydrogel	7.78 ± 0.12B	5.96 ± 0.13AB	4.18 ± 0.15AB	3.81 ± 0.09CD
	**Mean with irrigation**		**7.68 ± 0.14**	**5.93 ± 0.14**	**4.10 ± 0.14**	**3.88 ± 0.26**
	**Mean control**		**7.69 ± 0.12**	**5.89 ± 0.15**	**3.98 ± 0.14**	**3.47 ± 0.19**
	**Mean biostymulant**		**7.89 ± 0.17**	**6.22 ± 0.12**	**4.46 ± 0.16**	**3.87 ± 0.24**
	**Mean hydrogel**		**7.96 ± 0.14**	**6.12 ± 0.18**	**4.37 ± 0.15**	**3.60 ± 0.20**
	LSD Irrigation		0.16	0.18	0.36	0.26
	LSD antistress preparation		0.26	0.24	0.43	0.39
Denar	Without irrigation	Control	8.00 ± 0.16c	6.81 ± 0.11b	4.35 ± 0.16a	4.26 ± 0.34bc
		Biostymulant	8.33 ± 0.172d	7.20 ± 0.09d	5.23 ± 0.29b	5.18 ± 0.09d
		Hydrogel	8.461 ± 0.026d	7.00 ± 0.13c	5.31 ± 0.25b	5.08 ± 0.34d
	**Mean without irrigation**		**8.263 ± 0.137**	**7.00 ± 0.22**	**4.965 ± 0.532**	**4.84 ± 0.50**
	With irrigation	Control	7.56 ± 0.20a	6.48 ± 0.12a	4.108 ± 0.158a	3.85 ± 0.17a
		Biostymulant	7.84 ± 0.10bc	6.90 ± 0.11bc	4.518 ± 0.197a	4.50 ± 0.19c
		Hydrogel	7.68 ± 0.12ab	6.83 ± 0.07bc	4.557 ± 0.155a	4.06 ± 0.10ab
	**Mean with irrigation**		**7.69 ± 0.237**	**6.74 ± 0.20**	**4.39 ± 0.25**	**4.14 ± 0.33**
	**Mean control**		**7.78 ± 0.127**	**6.65 ± 0.13**	**4.23 ± 0.11**	**4.06 ± 0.22**
	**Mean biostymulant**		**8.08 ± 0.11**	**7.05 ± 0.16**	**4.88 ± 0.08**	**4.84 ± 0.13**
	**Mean hydrogel**		**8.07 ± 0.20**	**6.92 ± 0.16**	**4.93 ± 0.17**	**4.57 ± 0.15**
	LSD Irrigation		0.21	0.20	0.43	0.39
	LSD antistress preparation		0.43	0.22	0.51	0.53
LSD Cultivar			0.20	0.30	0.16	0.30

The values are means ± SD of twelve independent replicates. A-D (Gardena); a-d (Denar)—Means sharing the same letter in column are not significantly different (significance level of 0.05 using Tukey’s method).

Significant values are in [bold].

Under the influence of the irrigation treatment, the TS and RS content generally decreased in relation to that in the tubers derived from the plots on which no irrigation was applied (Table 2). A reduction in the TS and RS content in the Gardena cultivar tubers was by an average of 3.3% and 7.7%, respectively, while for the Denar cultivar, it was by an average of 3.7% and 13%, respectively. According to Bethke et al.^54^, Rykaczewska^55^, potatoes exposed to drought stress accumulate more sugars. Thompson et al.^56^, report that water deficiency changes the activity of key enzymes involved in carbohydrate metabolism, thereby RS the rate of their synthesis. However, in a study by Maggio et al.^57^, irrigation resulted in the accumulation of RS in the tubers of different potato cultivars, but only under organic cultivation, while under conventional cultivation, no such accumulation was observed. The highest levels of TS and RS were found in tubers derived from the plots where hydrogel without irrigation was applied (Table 2). Such a tendency was not noted only for the RS content in the Denar cultivar tubers in 2021. Kulikowski et al.^49^ reported that hydrogels, due to their high absorbency, can effectively store water not only from precipitation, fog, or dew but from any source. In general, the application of the biostimulant (Bio-Algeen S-90) and hydrogel had no significant effect on the content of ACH, AA or AC (Table 3). A different view was taken by Wszelaczyńska et al.^44^, Abou Chehade et al.^29^, Głosek-Sobieraj et al.^58^, and Caradonia et al.^52^, in whose studies the inoculation of plants with the biostimulant resulted in a significant reduction in the ACH content, and a significant increase in the content of AA and citric AC. Moreover, the irrigation applied in the current study significantly reduced the organic acid content in the tubers of the Gardena and Denar cultivars, which is consistent with the results obtained by other researchers^59–61^. On the other hand, Wang et al.^60^ report that the vitamin C content in potato tubers following irrigation initially increased and then decreased with the increased amount of water. Yang et al.^62^ are of a similar opinion, as they report that the relationship between the vitamin C content and the irrigation rate can be described by a quadratic equation.

**Table 3:** Post-harvest organic acid content of Gardena and Denar cultivars depending on irrigation and type of stress-reducing preparation.

Cultivar	Irrigation	Anti-stress preparation	Chlorogenic acid (mg kg^−1^ f.m.)		Askorbic acid (mg kg^−1^ f.m.)		Citric acid (mg kg^−1^ f.m.)	
			2020	2021	2020	2021	2020	2021
Gardena	Without irrigation	Control	300.9 ± 26.8A	320.38 ± 16.85B	208.2 ± 3.0B	196.1 ± 4.3AB	417.8 ± 12.3A-C	383.3 ± 2.6A-C
		Biostymulant	263.4 ± 41.4A	294.98 ± 22.34AB	213.9 ± 1.5C	201.8 ± 2.3BC	453.7 ± 38.4C	414.9 ± 34.7C
		Hydrogel	293.4 ± 19.6A	299.18 ± 21.46AB	214.9 ± 4.0C	205.0 ± 2.8C	445.3 ± 40.1BC	405.4 ± 47.2B-D
	**Mean without irrigation**		**285.9 ± 24.4**	**304.85 ± 27.12**	**212.3 ± 2.5**	**201.0 ± 1.3**	**392.4 ± 18.8**	**362.9 ± 16.3**
	With irrigation	Control	285.0 ± 55.5A	300.76 ± 3.99AB	200.9 ± 1.2A	190.3 ± 6.3A	382.5 ± 20.4A	351.0 ± 26.6A
		Biostymulant	256.5 ± 13.6A	271.14 ± 12.25A	205.2 ± 3.5AB	194.7 ± 2.1A	402.6 ± 10.6AB	375.7 ± 14.8A-C
		Hydrogel	271.9 ± 25.3A	288.01 ± 22.40A	203.5 ± 2.8AB	196.1 ± 1.7AB	392.0 ± 3.3A	361.8 ± 16.1AB
	**Mean with irrigation**		**271.14 ± 15.2**	**286.6 ± 15.2**	**203.2 ± 1.2**	**193.7 ± 2.1**	**438.9 ± 10.0**	**401.2 ± 12.4**
	**Mean control**		**293.0 ± 1.2**	**310.6 ± 13.2**	**204.5 ± 2.1**	**193.2 ± 1.7**	**400.1 ± 18.4**	**367.1 ± 25.2**
	**Mean biostymulant**		**259.9 ± 22.3**	**283.1 ± 16.4**	**209.5 ± 1.9**	**198.3 ± 2.4**	**428.2 ± 17.4**	**395.3 ± 21.1**
	**Mean hydrogel**		**282.7 ± 17.7**	**293.6 ± 20.1**	**209.2 ± 1.9**	**200.5 ± 3.1**	**418.7 ± 18.3**	**383.6 ± 28.5**
	LSD Irrigation		n.s.*	n.s	3.6	n.s	n.s	n.s
	LSD antistress preparation		n.s	n.s	7.0	n.s	n.s	26.6
Denar	Without irrigation	Control	267.2 ± 18.7c	299.2 ± 25.0b	217.2 ± 2.0bc	206.8 ± 1.9b	390.9 ± 19.2bc	321 ± 30.0ab
		Biostymulant	236.4 ± 8.20a-c	286.3 ± 23.1ab	221.0 ± 1.6 cd	206.4 ± 10.1b	397.1 ± 12.7c	373.6 ± 14.4c
		Hydrogel	240.4 ± 26.2a-c	289.0 ± 19.7ab	223.3 ± 3.2d	208.8 ± 3.0b	383.3 ± 16.9a-c	342.4 ± 21.4bc
	**Mean without irrigation**		**248.0 ± 17.2**	**291.5 ± 20.1**	**220.4 ± 1.2**	**207.3 ± 7.2**	**390.4 ± 17.2**	**345.7 ± 20.7**
	With irrigation	Control	257.3 ± 24.0bc	277.0 ± 37.9ab	209.0 ± 0.8a	193.5 ± 1.6a	357.2 ± 15.5a	296.8 ± 16.3a
		Biostymulant	220.3 ± 11.3a	250.1 ± 20.1a	215.9 ± 0.3b	201.1 ± 4.2ab	380.7 ± 9.7a-c	351.5 ± 18.5bc
		Hydrogel	226.9 ± 7.5ab	261.8 ± 33.4ab	215.0 ± 4.1b	203.2 ± 5.8b	367.3 ± 15.3ab	323.7 ± 5.8ab
	**Mean with irrigation**		**234.8 ± 18.1**	**263.0 ± 24.7**	**213.3 ± 1.5**	**199.3 ± 2.1**	**370.4 ± 14.6**	**324.0 ± 22.9**
	**Mean control**		**262.2 ± 12.5**	**288.1 ± 20.5**	**213.1 ± 1.7**	**200.1 ± 2.0**	**374.1 ± 17.7**	**308.9 ± 21.9**
	**Mean biostymulant**		**228.4 ± 14.3**	**268.2 ± 18.2**	**218.4 ± 1.9**	**203.8 ± 1.8**	**391.9 ± 21.6**	**362.5 ± 16.2**
	**Mean hydrogel**		**233.7 ± 17.2**	**275.4 ± 17.4**	**219.1 ± 2.0**	**206.0 ± 1.3**	**375.3 ± 22.4**	**333.1 ± 17.6**
	LSD Irrigation		n.s	n.s	3.6	n.s	n.s	n.s
	LSD antistress preparation		n.s	n.s	5.6	n.s	n.s	25.7
LSD Cultivar			18.2	16.7	3.8	4.3	19.0	21.2

*n.s. not significant.

The values are means ± SD of twelve independent replicates. A-D (Gardena); a-d (Denar)—Means sharing the same letter in column are not significantly different (significance level of 0.05 using Tukey’s method).

Significant values are in [bold].

**Figure 5:**
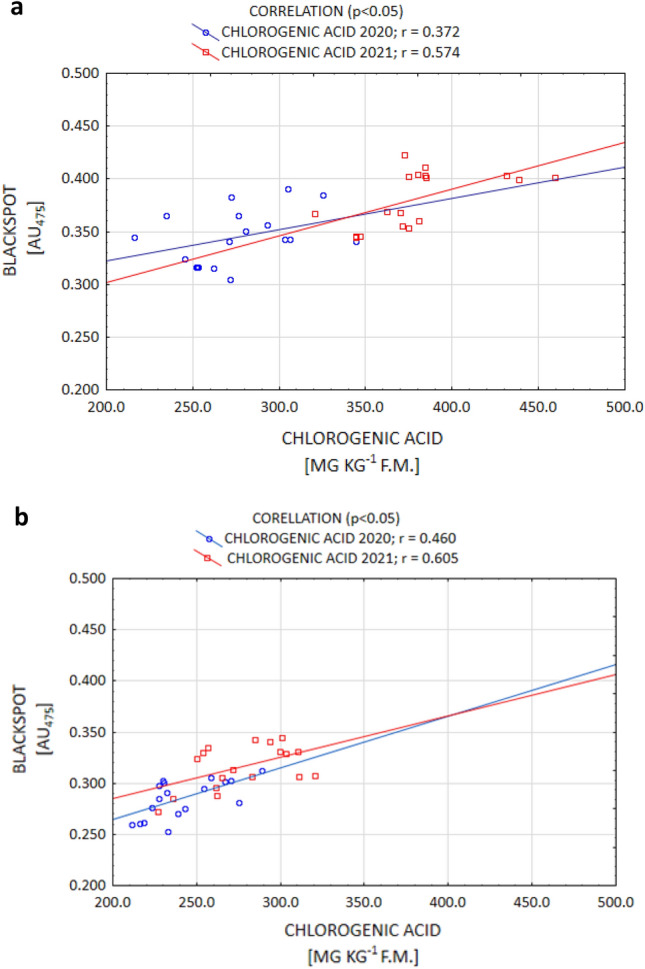
Post-harvest correlation between OP (blackspot) and ACH content of Gardena (**a**) and Denar (**b**) cultivars in 2020 and 2021 crop years.

**Figure 6:**
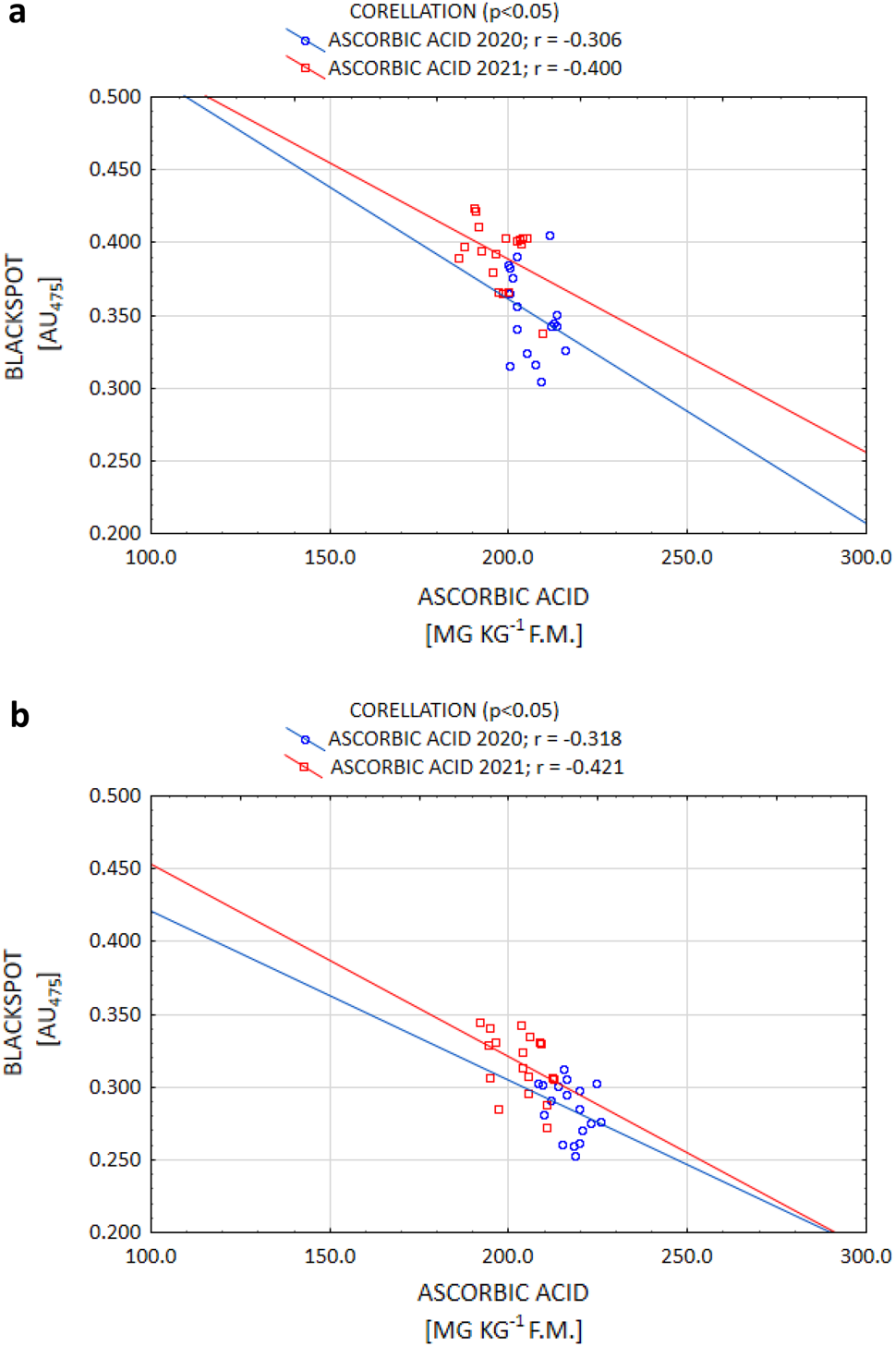
Post-harvest correlation between OP (blackspot) and AA content of Gardena (**a**) and Denar (**b**) cultivars in 2020 and 2021 crop years.

It is commonly known that of all the organic acids, ACH determines the susceptibility to enzymatic darkening the most by contributing to enhancing this process. On the other hand, the action of citric and AAs in this regard is weaker^9,17,35^. However, certain authors^35,52^ report that the ACH content is under genetic control, while the CA and AA contents are strongly influenced by the conditions during plant growth. The current study confirms that thesis, as a positive correlation between enzymatic darkening and ACH (and a negative correlation between darkening and AA) were demonstrated. Enzymatic darkening was determined, to the largest extent, by the ACH content for both the Gardena and Denar cultivars (Fig. 5a,b). The current study also confirmed a strong significant relationship between enzymatic darkening and AA for the Denar cultivar, and a weaker one in the Gardena cultivar, at a level *p* < 0.05 (Fig. 6a,b). No correlation, however, was noted between enzymatic darkening and the AC content. According to Rodríguez Galdón et al.^63^, AC, by forming complexes with oxidising metals, has a synergistic reducing effect with AA.

The tubers of the Gardena and Denar cultivars exhibited significantly greater susceptibility to enzymatic darkening following storage, as compared to the tests conducted immediately following the harvest (Table 4). Following storage, the potential oxidative value of the tubers increased by 15.8% for the Gardena cultivar and by 16.0% for the Denar cultivar (average values from the years of the study). It was pointed out that after long-term storage, the tubers of the Gardena cultivar changed the class of susceptibility to the oxidative darkening processes, according to the Dean’s table^32^, from moderately resistant to moderately susceptible (Table 1). Since the Gardena and Denar cultivars under study are in the group of table cultivars, they were stored under the conditions designated for this market destination, i.e. at a temperature of 4 °C. Many authors^16,17,64–66^ report that the most important factors determining the susceptibility to enzymatic darkening of the potato tuber flesh include the temperature and duration of storage. An additional factor may be the method of handling the raw material following the harvest. According to Adams & Brown^17^, Cabezas-Serrano et al.^64^, Murniece et al.^66^, the flesh of the tubers stored at lower temperatures for a long period darkens more intensely as compared to the tubers stored at higher temperatures and for a shorter period. On the other hand, Cabezas-Serrano et al.^64^, and Urbany et al.^65^ report that the change in the colour of the flesh of the potato tubers stored at low temperatures results from an enzymatic reaction that takes place following damage to the cell wall. Increased susceptibility to enzymatic darkening of the flesh following storage can also be due to adverse changes in the chemical composition of the tubers^17^.

**Table 4:** Percentage changes in OP (blackspot), content of TS, RS, and organic acids of Gardena and Denar cultivars after storage.

Cultivar	Irrigation	Anti-stress preparation	Blackspot (%)		Total sugars (%)		Reducing sugars (%)		Chlorogenic acid (%)		Askorbic acid (%)		Citric acid (%)	
			2020	2021	2020	2021	2020	2021	2020	2021	2020	2021	2020	2021
Gardena	Without irrigation	Control	+ 14.0	+ 17.4	+ 14.2	+ 9.3	+ 63.1	+ 64.7	+ 2.7	+ 12.9	-7.1	-7.0	+ 0.8	+ 4.3
		Biostymulant	+ 23.1	+ 18.1	+ 6.3	+ 0.4	+ 38.0	+ 54.8	+ 2.9	+ 13.7	-7.2	-6.8	+ 0.8	+ 2.2
		Hydrogel	+ 30.8	+ 17.7	+ 21.5	+ 4.1	+ 53.9	+ 57.9	+ 3.3	+ 13.8	-7.4	-7.0	+ 0.8	+ 5.3
	**Mean without irrigation**		** + 22.3**	** + 17.7**	** + 14.0**	** + 4.5**	** + 51.2**	** + 59.0**	** + 2.9**	** + 13.5**	**-7.3**	**-7.0**	** + 0.8**	** + 3.9**
	With irrigation	Control	+ 14.9	+ 13.3	+ 17.3	+ 14.1	+ 32.0	+ 52.7	+ 2.7	+ 13.4	-7.9	-7.7	+ 0.9	+ 4.8
		Biostymulant	+ 9.5	+ 13.7	+ 7.0	+ 2.0	+ 17.7	+ 29.4	+ 2.9	+ 13.9	-8.0	-7.6	+ 0.9	+ 6.3
		Hydrogel	+ 13.0	+ 4.6	+ 7.2	+ 14.9	+ 17.1	+ 44.4	+ 3.1	+ 14.6	-8.0	-7.9	+ 0.9	+ 2.2
	**Mean with irrigation**		** + 12.4**	** + 10.7**	** + 10.4**	** + 9.0**	** + 22.0**	** + 42.1**	** + 2.9**	** + 14.0**	**-8.0**	**-7.7**	** + 0.9**	** + 4.4**
	**Mean control**		** + 14.4**	** + 15.4**	** + 15.7**	** + 11.7**	** + 47.7**	** + 58.4**	** + 2.7**	** + 13.1**	**-7.5**	**-7.4**	** + 0.9**	** + 4.6**
	**Mean biostymulant**		** + 22.3**	** + 11.7**	** + 14.5**	** + 9.4**	** + 36.6**	** + 50.6**	** + 3.2**	** + 14.2**	**-7.7**	**-7.5**	** + 0.8**	** + 3.8**
	**Mean hydrogel**		** + 16.4**	** + 16.0**	** + 6.6**	** + 0.8**	** + 28.3**	** + 41.3**	** + 2.9**	** + 13.8**	**-7.6**	**-7.2**	** + 0.8**	** + 4.1**
	**Mean**		** + 18.9**	** + 15.1**	** + 12.7**	** + 6.7**	** + 40.8**	** + 52.6**	** + 2.9**	** + 13.7**	**-7.5**	**-7.2**	** + 0.8**	** + 4.1**
Denar	Without irrigation	Control	+ 23.0	+ 12.7	+ 18.6	+ 34.6	+ 70.5	+ 84.6	+ 2.8	+ 15.5	-4.2	-4.1	+ 0.6	+ 3.5
		Biostymulant	+ 14.7	+ 12.5	+ 25.8	+ 42.0	+ 57.7	+ 72.3	+ 3.2	+ 16.0	-4.3	-4.2	+ 0.7	+ 4.7
		Hydrogel	+ 32.9	+ 13.4	+ 6.2	+ 43.5	+ 33.6	+ 76.5	+ 3.3	+ 16.0	-4.5	-4.4	+ 0.7	+ 5.0
	**Mean without irrigation**		** + 23.3**	** + 20.5**	** + 16.9**	** + 40.1**	** + 53.0**	** + 77.4**	** + 3.1**	** + 15.8**	**-4.3**	**-4.8**	** + 0.7**	** + 4.4**
	With irrigation	Control	+ 16.3	+ 8.6	+ 25.8	+ 29.8	+ 53.1	+ 61.6	+ 2.6	+ 16.6	-4.7	-4.7	+ 0.8	+ 7.7
		Biostymulant	+ 21.1	+ 5.1	+ 19.3	+ 17.3	+ 34.5	+ 44.2	+ 3.0	+ 17.4	-4.7	-4.6	+ 0.8	+ 3.2
		Hydrogel	+ 24.8	+ 8.1	+ 5.0	+ 27.6	+ 21.0	+ 48.7	+ 3.2	+ 18.2	-4.9	-4.8	-0.8	+ 2.1
	**Mean with irrigation**		** + 12.8**	** + 7.3**	** + 16.6**	** + 24.9**	** + 35.7**	** + 51.2**	** + 3.0**	** + 17.4**	**-4.2**	**-4.7**	** + 0.2**	** + 4.2**
	**Mean control**		** + 19.7**	** + 10.7**	** + 22.1**	** + 32.3**	** + 62.1**	** + 73.7**	** + 2.7**	** + 16.0**	**-4.4**	**-4.4**	** + 0.7**	** + 5.5**
	**Mean biostymulant**		** + 29.0**	** + 10.8**	** + 5.6**	** + 35.7**	** + 27.7**	** + 63.5**	** + 3.2**	** + 17.0**	**-4.7**	**-4.6**	** + 0.0**	** + 3.6**
	**Mean hydrogel**		** + 17.9**	** + 8.9**	** + 22.7**	** + 29.8**	** + 47.0**	** + 59.8**	** + 3.1**	** + 16.7**	**-4.5**	**-4.4**	** + 0.7**	** + 4.0**
	**Mean**		+ 22.1	+ 10.1	+ 16.8	+ 32.5	+ 45.1	+ 64.6	+ 3.0	+ 16.6	-4.6	-4.5	+ 0.5	+ 4.4
**Mean cultivar**			+ 20.5	+ 12.6	+ 14.7	+ 19.6	+ 42.9	+ 58.6	+ 3.0	+ 15.1	-6.0	-5.9	+ 0.7	+ 4.2

These changes concern, *inter alia*, the content of sugars. After six months of storage, an increase was noted in the TS and RS content. The increase in the TS and RS content following long-term storage was much greater in the tubers of the Denar cultivar (by 24.6 Dean’s table and 54.3%, respectively), as compared to the Gardena cultivar (by 9.5 Dean’s table and 43.6%, respectively) (Table 6). A similar view was taken by Cabezas-Serrano et al.^64^, Alamar et al.^27^, Amjad et al.^67^, Morales-Fernández et al.^68^, and Zhang and Zhen-Xiang^69^, who believe that the accumulation of TS and RS during the storage of potatoes is influenced by genetic determinants. The above-mentioned researchers also report that the RS content following storage is influenced by the duration and conditions of storage, particularly the temperature. As the storage time increases, so does the RS content. A similar effect is achieved by low temperatures of 2–4 °C, at which the well-known phenomenon of cold-induced sweetening (CIS) occurs^67,69^.

The analysis of the results of the increase in the TS and RS content following storage indicates a large discrepancy between the results in this regard. The increase in the TS content ranged from 0.4% to as much as 43.5%, while for RS, the increase ranged from 17.1% to 84.6%. Such a large discrepancy between the results may be due to the different susceptibility of the cultivars to starch degradation in the sweetening process occurring at a low temperature of 4 °C. These results may also have been influenced by the factors applied during the growing season (high soil temperatures, transitory soil moisture deficits, and insufficient or excess nitrogen fertilization)^56^.

The increase in the RS content in the tubers following storage for the cultivars and years of the study was, on average, 49%, with the greatest increase (60.1%) noted for the tubers derived from the plots on which no irrigation was applied during the growing season (Table 4). Ohara-Takada et al.^70^, and Malone et al.^71^ pointed to a marked increase in the content of RS (glucose and fructose) during storage at low temperatures. This may be related to a process known as low-temperature sweetening^67,69–71^. According to Ohara-Takada et al.^70^, the increase in the RS content is related to the length of the storage period, as the authors demonstrated a four-fold increase in the RS content following long-term storage.

**Figure 7:**
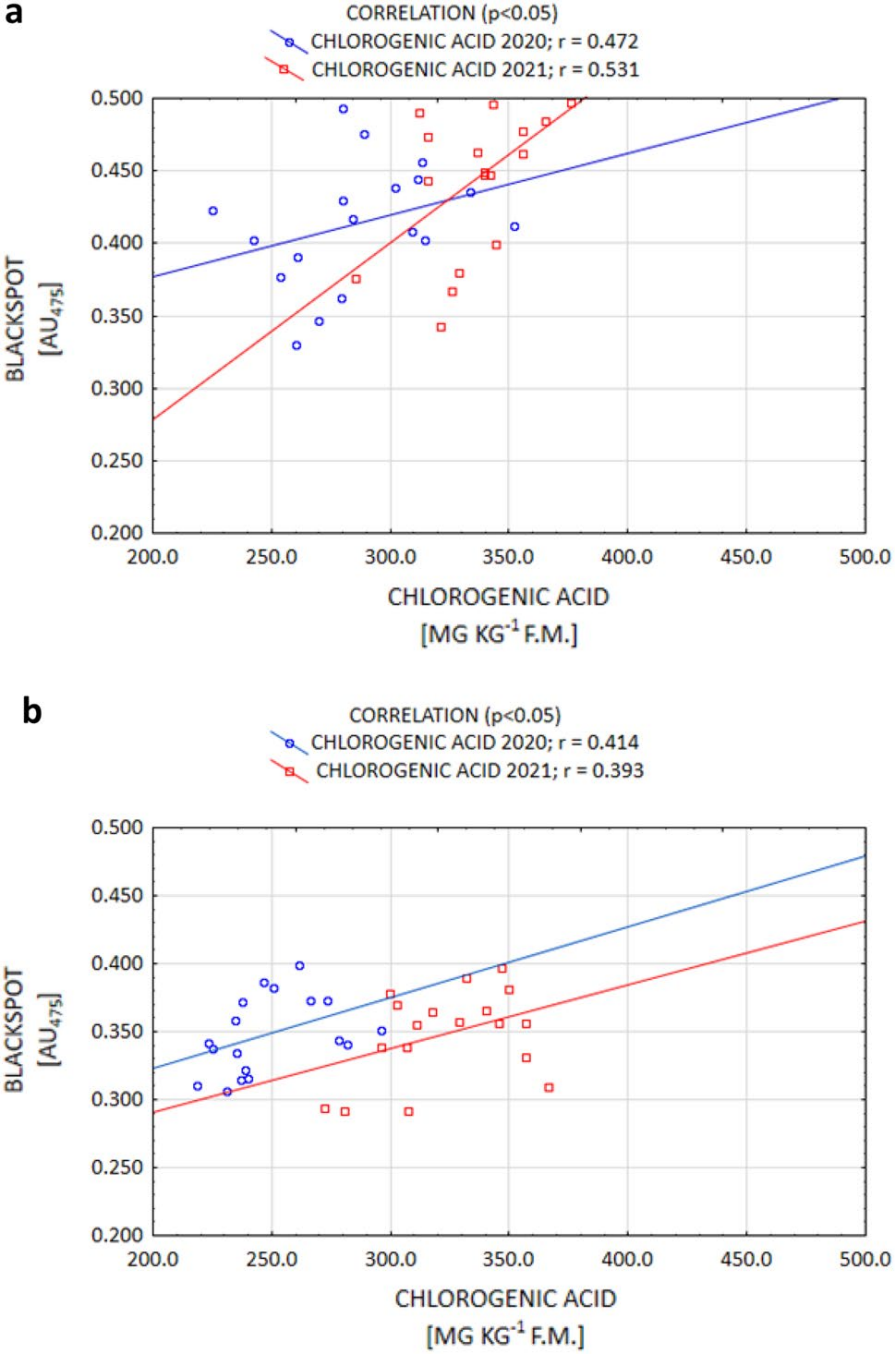
After-storage correlation between OP (blackspot) and ACH content of Gardena (**a**) and Denar (**b**) cultivars in 2020 and 2021 crop years.

**Figure 8:**
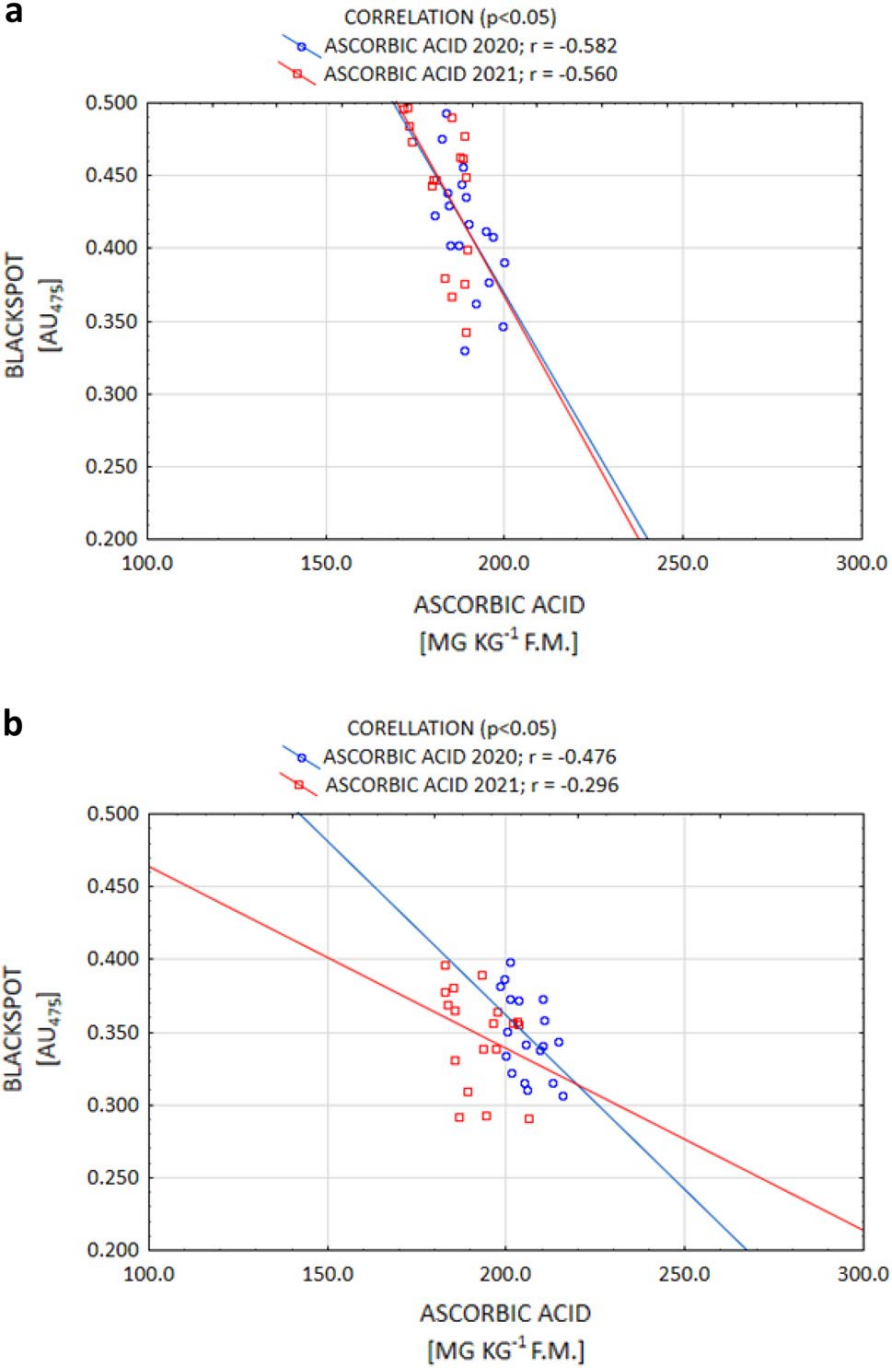
After-storage correlation between OP (blackspot) and AA content of Gardena (**a**) and Denar (**b**) cultivars in 2020 and 2021 crop years.

The change in the OP after the storage of tubers is also determined by the organic acid concentration. The results of the current study demonstrated that the ACH content increased following storage in both years of the study. In the year 2020, the increase in the ACH content was, on average, 3.0% for the cultivars, while in the year 2021, which was unfavourable for potato cultivation, it amounted to 13.7% for Gardena, and 16.6% for Denar (Table 4). According to Adams and Brown^17^, the increase in the ACH content during storage is due to its more rapid synthesis in relation to the depletion associated with the enzymatic browning process. However, as expected, in both years of the current study, the AA content decreased following storage. Such an effect was achieved for both the Gardena and Denar cultivars. Irrespective of the years of the study, the depletion was 7.5% for Gardena, and 4.5% for Denar (Table 4). As reported by Torres-Contreras et al.^72^, and Pobereżny et al.^16^, storage always results in an increase in the ACH content and a loss of AA. In addition, Adams and Brown^17^, and Wszelaczyńska et al.^44^, proved that the OP of potatoes following storage was positively correlated with an increase in the ACH content, and in a study by Mondy and Munshi^73^, it was negatively correlated with the depletion of AA. Current research has confirmed such a relationship. In both 2020 and 2021, a positive correlation was demonstrated between the OP and ACH (Fig. 7), while a negative correlation was demonstrated between the OP and AA (Fig. 8). According to Mondy and Munshi^73^, and Adams and Brown^17^, enzymatic browning of potatoes is correlated with the biochemical transformations associated with the utilisation of tyrosine. On the other hand, the magnitude of transformations utilising tyrosine is determined to a greater extent by the interaction of the concentrations of PPO, ACH, and AA than by a single parameter.

The current study demonstrated a slight increase of approx. 0.6% (an average for the cultivars) in the AC content in the tubers following storage in 2020, and by 4.2% in 2021 (Table 4). Similar results were obtained by Wszelaczyńska et al.^44^ in a study in which an increase in the AC content was noted following long-term storage of the tubers of cultivars of different earliness. For table cultivars, the increase was by an average of 4.0%, while for the cultivars intended for processing, by an average of 2.0%. On the other hand, Drapal et al.^74^ achieved a major increase in the AC content in tubers following storage.

## Conclusions

The OP of potato tubers was demonstrated to be significantly influenced by the conditions of the growing season in interaction with both genotypic and technological variability. The flesh of the tubers of the Denar cultivar was characterised by a lower susceptibility to enzymatic darkening. In general, the anti-stress factors applied during potato cultivation significantly reduced the OP of the tubers. Long-term storage contributed to changes in the sugar and organic acid contents in potatoes. After six months, the potato tubers exhibited a greater susceptibility to enzymatic darkening, which was significantly influenced by the increase in the content of both TS and RS and ACH. The Gardena cultivar changed the class of susceptibility to darkening from moderately resistant to moderately susceptible, which shows that it is more sensitive to long-term storage. The change in the OP value following storage was also influenced by the factors applied during the potato growing season.

The current knowledge in the field of environmental protection and the obtained results indicate that research should be conducted in order to precisely determine the doses of the hydrogel and biostimulant in terms of the consumption value of the potato, including the tendency to darkening the tubers as a feature determining the choice by the consumer. In addition, it is necessary to conduct research aimed at evaluating the effect of anti-stress preparations in the cultivation of a larger number of edible potato cultivars belonging to one earliness group.

## Material and methods

### Study material

This study tested potatoes of two edible cultivars, i.e. Gardena and Denar. Gardena is a medium-early cultivar of a general-to-slightly-floury table type (B-BC), while Denar is a very early cultivar of the general table and salad type (AB). The flesh of the tubers of these varieties is pale yellow in colour. The Gardena cultivar is characterised by light pink, very delicate skin, and high resistance to the late blight *Phytophthora infestans* (7–8). On the other hand, Denar is one of the cultivars very susceptible to late blight.

### Ethical approval

All procedures with plants were conducted in accordance with the guidelines and regulations.

### Field experiment

The field experiment was conducted in the years 2020 and 2021 in Mazowieckie Voivodeship at the Station of the Plant Breeding and Acclimatisation Institute (IHAR) in Jadwisin (52°47’N, 21°04’E) situated in central Poland.

According to the IUSS Working Group WRB^75^, the soil used in the experiment was predominantly luvisol (LV). The experiments were located on light soil with a mechanical composition of loamy sand, valuation class 5, good rye complex, with low humus content (Table 5). The growing season of 2020 can be described as wet and moderately warm. However, the year 2021, with its very high humidity and moderate heat, was less favourable for potato cultivation (Table 6).

**Table 5:** Chemical properties of the soil at the location of the field experiment of IHAR-PIB o/Jadwisin.

Years	Humus (%)	Reaction (pH in KCl)	Macronutrient content (mg kg^−1^ g soil)			Micronutrient content (mg kg^−1^ soil)				
			P_2_O_5_	K_2_O	Mg	Mn	Zn	Cu	B	Fe
2020	0.92	5.0	196	130	27	108.0	4.5	4.0	0.50	544.0
2021	0.77	5.0	194	115	22	88.2	3.5	2.5	0.50	568.3

**Table 6:** Arrangement of weather conditions in the years of the study based on the meteorological station of IHAR-PIB o/ Jadwisin.

Years	Months						
	IV	V	VI	VII	VIII	IX	IV-IX
Total rainfall (mm)							
2020	5.6	65.3	113.8	40.4	120.7	51.8	397.6
2021	37.8	69.5	97.2	124.2	120.4	37.5	486.6
Standard*	34.0	57.0	75.0	75.0	61.0	49.0	351.0
The average air temperature (°C)							
2020	8.8	11.6	18.7	19.0	20.1	15.5	14.8
2021	6.9	12.7	20.1	21.9	17.2	13.5	15.4
Standard*	8.1	13.7	16.8	18.6	18.1	13.3	14.7

*Years 1967–2019.

For the experiment in Jadwisin, we used two edible potato cultivars: ‘Denar’ (very early): the average yield determined by COBORU in registered studies was 43.7 t ha^−1^ with a starch content in tubers of approx. 11.7%, culinary type general to salad (AB) with a fairly good taste of 7.2*, the tubers have a light yellow-yellow flesh color and are quite large (8*), oval or round-oval shape with quite shallow eyes (6.9*), little darkening (8.6–8.0*) and ‘Gardena’ (medium early): the average yield determined by COBORU in registered studies was 47.9 t ha^−1^ with a starch content in tubers of approx. 13.4%, culinary type general to slightly floury (B-BC), of general culinary type to slightly floury (B-BC), with a fairly good taste of 6.7*, the tubers have a light yellow flesh color and are quite large (7–8*), oval or even elongated shape with very shallow eyes (7.9*); *on a 9-point scale according to COBORU*). Potato cv. Denar is a very popular edible potato on the market in Poland, while Gardena is a cv. highly resistant to *Phytophtora infestans* with high utility value and the forecrop in potato cultivation was triticale. The potatoes were planted in the 3rd ten-day period of April at a spacing of 0.75 × 0.33 m, and harvested in the 3rd ten-day period of September (cv. Denar—20.09.2020 and 21.09.2021; cv. Gardena—29.09.2020 and 30.09.2021). The experimental design was a split-split-plot (2–2-3), randomized block with three replications and a single plot size of 15 m^2^. The experiment compared two treatments: one without irrigation (control), and another one with irrigation, on which sprinkler irrigation was applied. Reel sprinkler with irrigation console IRTEC 82G/D6 450 made in Italy, irrigation console range 30 m. The 20 mm spray nozzle, 3 atmosphere pressure at the end. The irrigation rates were determined by the balance method, and soil moisture measurements were taken using a TDR moisture sensor. In the growing season of 2020, two irrigation treatments were applied (a total of 35.7 mm), while in 2021, one irrigation treatment of 13 mm was applied. The following were applied on each plot (anti-stress preparations): without anti-stress preparations (control), foliar application of a biostimulating preparation ‘Bio-Algeen S90’ (Shulze & Hermsen GmbH, Dahlenberg, Germany) in an amount of 1.5 dm^3^ ha^−1^ in each of the three treatments at the BBCH stages of 30 (main stem elongation), 59 (inflorescence emergence—the final development stage), and 70 (the initial stage of fruit development), and a soil application of hydrogel (supersorbent) at a rate of 360 kg ha^−1^ during the planting. The Neptun 15 knapsack sprayer (Kwazar, Poland) was used for spraying BioAlgeen S90—swirl tip, fine spray, working pressure (MPa) 0.1–0.15. Organic microfertiliser ‘Bio-Algeen S90’ is a natural, plant-based fertiliser from an extract derived from marine algae (*Ascophyllum nodosum*) and seaweed. The preparation contains 90 groups of including amino acids, vitamins, alginic acid and other active ingredients. The content of the most important elements is as follows: nitrogen (N)—0.02%, phosphorus (P_2_O_5_)—0.006%, potassium (K_2_O)—0.096%, calcium (CaO)—0.31%, magnesium (MgO)—0.021%, boron (B)—16 mg kg^−1^, iron (Fe)—6.3 mg kg^−1^, copper (Cu)—0.2 mg kg^−1^, manganese (Mn)—0.6 mg kg^−1^, zinc (Zn)—1.0 mg kg^−1^. The preparation also contains molybdenum, selenium and cobalt. The fertiliser is 100% organic and not harmful to humans or animals. It can be used in combination with other plant protection products, as it does not change their effects. The application of the product requires no waiting period. The hydrogel (Rolmarket, Poland) preparation is in the form of granules (0.35 mm–1.10 mm); pH 6–10; 1 g of hydrogel absorbs from 200 to 600 g of water.

All mineral fertiliser doses were applied in the spring prior to planting the potatoes in amounts taking into account the nutritional requirements of the crops in each year of the study:

- mineral nitrogen, at a rate of 100 kg N ha^−1^ (urea 46%, N),
- phosphorus at a rate of 60 kg P_2_O_5_ ha^−1^ (enriched superphosphate 40%, P_2_O_5_),
- potassium at a rate of 120 kg K_2_O ha^−1^ (potassium salt 60%, K_2_O)

Protection against pathogens was carried out using pesticides. Weeds were controlled by applying a ridging plough with chains twice for emerging potato plants. Herbicides: immediately before emergence, after the final ridging, metobromuron (Proman 500 SC)^I,II^ was applied at a rate of 4 dm^3^ ha^−1^ (BBCH 09), while following the emergence of potato plants, rimsulfuron (Titus 23 WG)^I,II^ was applied at a rate of 60 g ha^−1^, and ethoxylated isodecyl alcohol adjuvant (Trend 90 EC)^I,II^ was applied at a concentration of 0.1% (BBCH 29). Fungicides: protection against the late blight was carried out using the following chemical preparations: metalaxyl-M and mancozeb (Ridomil Gold MZ Pepite 67,8 WG)^I^ 2.5 kg ha^−1^ (BBCH 50); mandipropamide and difenoconazole (Carial Star 500 SC)^I,II^ 0.6 l ha^−1^ (BBCH 69); dimethomorph and mancozeb (Acrobat MZ 69 WG)^I,II^ 2 kg ha^−1^ (BBCH 75); fluazinam (Banjo 500 SC)^II^ 0.4 dm^3^ ha^−1^ (4 treatments in 2020, and 3 treatments in 2021) (BBCH 89). Insecticides: imidacloprid (Nuprid 200 SC)^I^ 0.15 dm^3^ ha^−1^ (BBCH 39); chlorantraniliprole (Coragen 200 SC)^I^ 62.5 cm^3^ ha^−1^ (BBCH 50); thiacloprid (Calipso 480 SC)^I^ 0.1 dm^3^ ha^−1^ (BBCH 85); spinoset (Spintor 240 SC)^I,II^ 0.15 dm^3^ ha^−1^ (BBCH 65); acetamiprid (Carnadine 200 SL)^II^ 0.18 dm^3^ ha^−1^ (BBCH 50); (2020^I^, 2021^II^).

Laboratory tests were conducted immediately after harvesting the potatoes at the Faculty of Agriculture and Biotechnology, Bydgoszcz University of Science and Technology, and following long-term storage (for six months). 10 kg samples were stored in chambers where constant conditions were maintained throughout the period, in line with the market destination of the potatoes: a temperature of 4 °C, and relative air humidity of 95%.

### Laboratory tests

There were four laboratory replicates for each combination.

### Freeze-drying of fresh potato tubers

For the freeze-drying process, medium-sized tubers were washed and cut into pieces. The tuber samples (200 g) were initially frozen in a Whirlpool AFG 6402 E-B freezer (Italy) to a temperature of − 22 °C. Sublimation drying was conducted in a CHRIST ALPHA 1–4 LSC device (Germany) at the following freeze-dryer operating parameters: a condenser temperature of 55 °C, vacuum 4 kPA at 20 °C. Potato tuber samples were dried to a constant weight. The final moisture content in the material was less than 2%. Drying was continued for 24 h.

### Quality characteristics of potato tubers

#### TS and RS analysis

The procedure for determining TS and RS according to the G-26 TEST. The TS and RS content was measured by the DNP method^76^. The method consisted in placing a homogeneous sample (1 g of freeze-dried potato) in a 250 cm^3^ volumetric flask, adding 150 cm^3^ of distilled water, and shaking vigorously for 360 s, followed by filtering through Whatman filter paper (International Limited, Kent, UK). In order to determine the RS content, after filtration, 1 cm^3^ was transferred to a test tube with a diameter of 2 10^−2^ m, 3 cm^3^ of the DNP solution (Sigma Aldrich, St. Louis, MO, USA) were added, and the contents of the tube were shaken vigorously for 10 s, and heated in a boiling water bath for 6 min. The test tube was then cooled in cold water, and absorption was measured in 1 × 1 10^−3^ m thick cuvettes at a wavelength of 600 nm using a SHIMADZU UV-1800 spectrophotometer (UV–Vis Spectral Photometer System, Japan). The spectrophotometer was reset to zero using distilled water. A calibration curve was prepared using glucose. In order to determine the TS content, 40 cm^3^ of the filtrate was measured and placed in a 100 cm^3^ conical flask. The solution in the flask was then acidified by adding two drops of concentrated HCl. The conical flask with the solution was covered with aluminium foil and heated in a boiling water bath for 30 min. The samples were cooled in cold water, and 2–3 drops of concentrated NaOH were added to neutralise the solution. For the determinations, a 1 cm^3^ sample was taken, 3 cm^3^ of DNP reagent was added, and the procedure for determining the RS content was followed.

#### Determination of AA

The AA content was determined according to Kapur et al.^77^. Five grams of the freeze-dried potato sample were homogenised with 25 cm^3^ of metaphosphoric acid/acetic acid, transferred quantitatively to a 50 cm^3^ volumetric flask, and shaken gently. The sample was then diluted to the mark with the metaphosphoric acid/acetic acid solution, and the whole sample was thoroughly mixed. The obtained solution was filtered through Whatman filter paper (International Limited, Kent, UK) and centrifuged at 4000 rpm for 15 min (Hettina Zentrifugen, Rotina 420 R, Germany). The supernatant was then used for spectrophotometric determination (UV-1800, UV Spectrophotometer System, Japan) of the AA content in the samples. The procedure: 0.23 cm^3^ of 3% bromine water was added to 4 cm^3^ of the centrifuged sample solution in order to oxidise AA to dehydroAA, and then 0.13 cm^3^ of 10% thiourea was added to remove excess bromine. Subsequently, 1 cm^3^ of 2,4-DNPH solution was added to form an osazone. All standards, samples, and blank solutions were maintained at 37 °C for three h in a thermostatic bath. The samples were then cooled in an ice bath for 30 min, and 5 cm^3^ of cooled 85% H_2_SO_4_ were added while stirring continuously. Consequently, the absorbance of the coloured solution was measured in cuvettes with a thickness of 1 × 1 10^−3^ m at a wavelength of 521 nm. A calibration curve within the concentration range of 0–1000 mg kg^−1^ was prepared using an AA solution (POCH S.A., Gliwice, Poland).

#### Determination of ACH

The ACH content was determined by the colorimetric method by Griffiths et al.^78^, using sodium nitrate for the reaction. Freeze-dried potato powder (200 mg) was placed in a centrifugal flask and vortexed with 2 cm^3^ of urea (0.17 M) and acetic acid (0.10 M). Subsequently, 1 cm^3^ of sodium nitrate (0.14 M) and 1 cm^3^ of sodium hydroxide (0.5 M) were added and vortexed again, and the solution was then incubated at room temperature for 2 min. The obtained suspension was centrifuged at 2250 rpm. for 10 min (Hettina Zentrifugen, Rotina 420 R, Germany). An aliquot of the supernatant was collected, and the absorbance of the red-solution-coloured complex formed was measured in cuvettes with a thickness of 1 × 1 10^−3^ m at 510 nm (using a SHIMADZU UV-1800 spectrophotometer, UV–Vis Spectral Photometer System, Japan). A calibration curve was prepared by using different ACH concentrations.

#### Determination of AC

The AC content was determined by the colorimetric method by Silva et al.^79^. Each measurement used 5 g of freeze-dried potato, which was placed in a 100 cm^3^ volumetric flask, to which 50 cm^3^ of distilled water was added, and the whole sample was shaken for 60 min. It was then made up to 100 cm^3^ with distilled water, vortexed again, and filtered through Whatman filter paper (International Limited, Kent, UK). Following this, 8 cm^3^ of the filtrate was mixed with 42 cm^3^ of 5% trichloroacetic acid (TCA). The mixture was then heated in a water bath at 80 °C for one hour and then centrifuged at 2500 rpm for five min. 0.5 cm^3^ of the centrifuged sample was pipetted into a dry test tube, and 4 cm^3^ of anhydrous acetic anhydride was added. The test tubes with the samples were capped and placed in a water bath at 60 °C for ten min. 0.5 cm^3^ of pyridine was added to each test tube, which was capped again and placed in a water bath at 60 °C for 40 min. The test tubes were cooled in tap water, and the absorbance was measured at a wavelength of 420 nm using a SHIMADZU UV-1800 spectrophotometer (UV–Vis Spectral Photometer System, Japan). The blank comprised 0.5 cm^3^ TCA top, to which acetic anhydride and pyridine were added sequentially. A calibration curve was prepared in 5% TCA with 15–400 mg aliquots of AC cm^−3^.

#### Determination of OP

The analysis was conducted by the colorimetric method^32^. Equal aliquots of 25 g each from fresh potato tubers (from the top, stolon, and central part of the plant) were homogenised in a laboratory blender (BOSCH, model MSM67170, BSH GmbH, Germany) for 60 s with 25 cm^3^ of 0.02 M phosphate buffer. The homogenate was left to oxidise for 24 h in a dark place. Sealed samples were maintained at a temperature of 20 °C. The samples were filtered through a Whatman filter paper (International Limited, Kent, UK). Following this, the samples were centrifuged at 12,000 rpm. Before photometric measurements, the samples were diluted at a ratio of 1:3. Absorbance was measured at 475 nm using a SHIMADZU UV-1800, UV–Vis spectrophotometric system (Japan). The blank comprised 0.02 M phosphate buffer. The results are an average value from three measurements and are presented as absorbance units (AU_475_) at 475 nm.

### Statistics

The obtained results were analysed using Statistica 13.1 software (StatSoft, Tulsa, OK, USA). The data were verified for normality of distribution the Shapiro–Wilk test (were transformed when required by either sqrt(x + 1) or ln(x + 1)) and homogeneity of variance, and the average values obtained in individual groups were subjected to two-way ANOVA (analysis of variance) at a significance level of 0.05 using Tukey’s method. Each year of research was analysed separately because the years of research were characterized by different weather conditions. Also, each cultivar was analysed separately because potato cultivars belonged to different earliness groups. The values were presented as averages with standard deviations (SD). Categorised 3W diagrams were used for data subsets in order to present the results. To identify the relationship between the distinguishing qualitative features under study, Spearman's rank correlation coefficients were determined at *p* = 0.05.

## Data Availability

The datasets used and/or analysed during the current study are available from the corresponding author(s) on reasonable request.
